# Ultra-High Performance Liquid Chromatography-High Resolution Mass Spectrometry and High-Sensitivity Gas Chromatography-Mass Spectrometry Screening of Classic Drugs and New Psychoactive Substances and Metabolites in Urine of Consumers

**DOI:** 10.3390/ijms22084000

**Published:** 2021-04-13

**Authors:** Emilia Marchei, Maria Alias Ferri, Marta Torrens, Magí Farré, Roberta Pacifici, Simona Pichini, Manuela Pellegrini

**Affiliations:** 1 National Centre on Addiction and Doping, Istituto Superiore di Sanità, V.Le Regina Elena 299, 00161 Rome, Italy; roberta.pacifici@iss.it (R.P.); simona.pichini@iss.it (S.P.); manuela.pellegrini@iss.it (M.P.); 2 Drug Addiction Program, Institut de Neuropsiquiatria i Addicions, Institut Hopsital del Mar d’Investigacions Mèdiques (INAD-IMIM), Parc de Salut Mar, 08003 Barcelona, Spain; malias@imim.es (M.A.F.); mtorrens@parcdesalutmar.cat (M.T.); 3 Department of Psychiatry and Pharmacology, Therapeutics and Toxicology, Universitat Autònoma de Barcelona, 08193 Cerdanyola del Vallés, Spain; mfarre.germanstrias@gencat.cat; 4 Clinical Pharmacology Unit, Hospital Universitari Germans Trias i Pujol and Institut de Recerca GermansTrias i Pujol (HUGTiP-IGTP), 08916 Badalona, Spain

**Keywords:** classic drugs of abuse, new psychoactive substances (NPS), novel synthetic opioids (NSO), urine, liquid chromatography, high-resolution mass spectrometry, gas chromatography-mass spectrometry

## Abstract

The use of the new psychoactive substances is continuously growing and the implementation of accurate and sensible analysis in biological matrices of users is relevant and fundamental for clinical and forensic purposes. Two different analytical technologies, high-sensitivity gas chromatography-mass spectrometry (GC-MS) and ultra-high-performance liquid chromatography-high-resolution mass spectrometry (UHPLC-HRMS) were used for a screening analysis of classic drugs and new psychoactive substances and their metabolites in urine of formed heroin addicts under methadone maintenance therapy. Sample preparation involved a liquid-liquid extraction. The UHPLC-HRMS method included Accucore™ phenyl Hexyl (100 × 2.1 mm, 2.6 μm, Thermo, USA) column with a gradient mobile phase consisting of mobile phase A (ammonium formate 2 mM in water, 0.1% formic acid) and mobile phase B (ammonium formate 2 mM in methanol/acetonitrile 50:50 (*v*/*v*), 0.1% formic acid) and a full-scan data-dependent MS2 (ddMS2) mode for substances identification (mass range 100–1000 *m*/*z*). The GC-MS method employed an ultra-Inert Intuvo GC column (HP-5MS UI, 30 m, 250 µm i.d, film thickness 0.25 µm; Agilent Technologies, Santa Clara, CA, USA) and electron-impact (EI) mass spectra were recorded in total ion monitoring mode (scan range 40–550 *m*/*z*). Urine samples from 296 patients with a history of opioid use disorder were examined. Around 80 different psychoactive substances and/or metabolites were identified, being methadone and metabolites the most prevalent ones. The possibility to screen for a huge number of psychotropic substances can be useful in suspected drug related fatalities or acute intoxication/exposure occurring in emergency departments and drug addiction services.

## 1. Introduction

A new psychoactive substance (NPS) is defined as “a new narcotic or psychotropic drug, in pure form or in preparation, that is not controlled by the United Nations drug conventions, but which may pose a public health threat comparable to that posed by substances listed in these conventions” [1].

In Europe, seizures of NPS mainly concern synthetic cannabinoids which together with synthetic cathinones account for more than 70% of NPS seizures [2]. Nevertheless, the more recent and most toxic NPS showed to be the novel synthetic opioids (NSOs). Since 2009, 57 new NSOs have been detected on Europe’s drug market [2]. Several NSOs were originally synthesized by pharmaceutical companies in their research for analgesic drugs as compounds with a similar chemical structure to natural opiates without addictive properties, but their toxicity or abuse potential posed a very high risk of poisoning to consumers. Whereas some of them were then marketed as prescription drugs, some others were eliminated from the licit market and some others were chemically modified to exclusively enter illicit market [3,4,5].

The chemical variety of NSOs, ranging from several illicit analogs of fentanyl and derivatives to newly synthesized molecules, make their identification extremely difficult and need the investigation of qualified analysts/toxicologists [6].

Since NSOs and particularly fentanyl-related compounds are active in very low doses, due to their potency and many users are unknowingly consuming these as adulterants in products sold as heroin, or as pain killers [7,8], parent drugs and metabolites are present in biological material at extremely low concentrations. One consequence of this is that they may escape detection because routine testing of these drugs is rarely performed and requires dedicated analytical methods with sufficiently high sensitivity and specificity [9].

The 2020 COVID-19 pandemic has transformed daily life and the different intensity of the lockdown across countries showed important consequences on drug users. The legal restrictions modified their ability to access classic illicit drugs (e.g., heroin, cocaine, cannabinoids) and shifted consumptions towards prescription psychoactive drugs, frequently available at home or from the use of psychoactive recreational NPS (e.g., synthetic cathinones, synthetic cannabinoids, phenethylamines to narcotic analgesics such as NSOs or to anxiolytics such as new benzodiazepines [10,11]. Nine new uncontrolled NSOs have been reported during 2020 [12]and the global shortage of heroin due to pandemic may have forced regular users to take other substances with similar effects, such as fentanyl analogs and NSOs [13].

In 2018 the JUSTSO project (analysis, dissemination of knowledge, implementation of Justice and special tests of new synthetic opioids), funded by European Commission, intended to evaluate, test profile and feedback into education and prevention, knowledge related to the NSO currently used in Europe, their nature, effects and associated harm [14].

Our main involvement in the project was to develop and validate analytical methodologies for the screening analysis of NSO and their metabolites, together with all other possible psychoactive drugs in urine samples of drug users collected in different settings (detoxification units, methadone maintenance clinics, drug addiction services, etc.).

Targeted/untargeted screening workflows based on gas or liquid chromatography coupled with mass spectrometry or tandem mass spectrometry (GC-MS, LC-MS and LC-MS/MS, respectively) play a central role in the daily activities of analytical laboratories operating in clinical and forensic toxicology. Specifically, urinalysis with multiple analytical technologies can increase the number of licit and illicit drugs band metabolites with different physicochemical properties that can be determined [15,16,17,18,19,20,21,22,23]. New pharmacologically active substances, both licit and illicit, are constantly being introduced and this occurrence has increased demand for new MS solutions that go beyond conventional GC-MS and LC-MS/MS. High-resolution mass spectrometry (HRMS) enables determination of the exact molecular formula (<5 ppm mass error) that can be useful for presumptive assignment of unknowns in general toxicology screenings [18].

Few previous studies performed in this field used one or more than one analytical tool for identification of a high number of unreported psychotropic substances in biological matrices of users.

Ultra-high performance liquid chromatography-tandem mass spectrometry (UHPLC-MS/MS) methodology has been applied not only to detect, but also to quantify 87 NPS and 32 classic illicit drugs and their metabolites in hair and nails [16] and 77 among the most abused NPS in blood, urine and oral fluid [17]. These two assays used only one type of instrument, but required the availability of all the pure standards of analytes under investigation for their quantification. Others screening methods coupled LC or GC with detection methods as time-of-flight mass spectrometry for analytical determination of NPS in seized samples [19] or in serum of consumers [20]. Moreover, to solve a complex toxicological fatal case due to NPS, several different analytical methodologies, including 1H nuclear magnetic resonance (NMR), GC–MS and UPLC–MS/MS to examine unambiguously seized material and biological fluids [21].

Finally, a combination of last generation GC-MS and UHPLC-HRMS has been recently employed by our investigation group to determine a selection of synthetic cannabinoids in oral fluid of consumers. Specifically, GC-MS has proven useful to identify and quantify parent compounds whereas UHPLC-HRMS also confirmed the presence of their metabolites in oral fluid [22].

Using the same combination of analytical methodologies, we hereby propose a screening method for urinalysis of principal NSOs, classical drugs of abuse and other NPS with main metabolites using a fast sample extraction.

## 2. Results

### 2.1. GC-MS and UHPLC-HRMS Methods

A simple and selective screening analysis with simultaneous use of high-sensitivity GC-MS and UHPLC-HRMS was applied for the identification of classic drugs of abuse, new psychoactive substances and metabolites in urine of drug addicts. The extraction procedure was tested with above reported fortified urine samples using different solvents. The mixture of chloroform and isopropanol has been found as the best compromise for the extraction of drugs and with acceptable signal-to noise ratio in an analytical screening, optimizing the extraction times and costs. Furthermore, even if the total analysis time was not short (each chromatographic run was completed in 32 min in GC/MS and 15 min for UHPLC-HRMS) the combined use of two instruments allowed to screen with a high percentage of compounds matched several different substances.

The characteristic retention times and monitored *m*/*z* ions used for the identification of mostly found substances monitored in urine samples are reported in Table 1.

The results obtained by screening proficiency urine testing samples from UNODC International Quality Assurance Program and those from in “NPS-LABVEQ” project showed an excellent agreement (98% agreement as screened substances) between substances declared and those found in the samples. Since these latter substances were analyzed at a concentration of 1 ng/mL urine with a signal to noise ratio, calculated at the baseline, always higher than 10, we could assume that our methodologies could screen substances present in concentrations equal or above 1 ng/mL. Moreover, from the analysis of blank urine no additional peaks due to endogenous substances, which could have interfered with the detection, were observed.

### 2.2. Methods Application

Drug screening applied to 296 former heroin addicts under methadone maintenance therapy urine disclosed the presence of different psychoactive prescription drugs, classical drugs of abuse, NSO, NPS and their metabolites. The presence of a certain drug and/or metabolites was confirmed only if both methodologies identified the molecules, which occurred in 95% cases.

**Figure 1:**
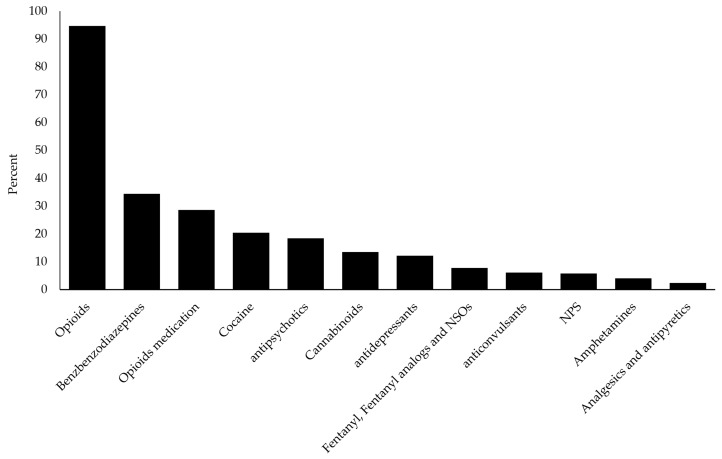
Percentage plot of classic drugs and new psychoactive substances found in 296 urine samples from former heroin users at methadone maintenance clinics and drug addiction services.

Pharmaceuticals like benzodiazepines, antidepressants, antipsychotics, anticonvulsants and opioids were detected. Drugs of abuse (opioids, amphetamines, cocaine and cannabinoids), NPS (synthetic cannabinoids, synthetic cathinones), fentanyls, NSO and other drug classes were also found. The frequency of different drug classes found in urine samples using the developed GC-MS and LC–HRMS screening methods is reported in Figure 1.

The most frequent found substances (about 90%) were methadone and its metabolites, 2-ethylidene-1,5-dimethyl-3,3-diphenylpyrrolidine (EDDP) and 2-ethyl-5-methyl-3,3-diphenylpyrroline (EMDP). Urine samples resulted positive also to benzodiazepines (mainly Clonazepam, Diazepam and their metabolites), antipsychotics (principally Risperidone, Quetiapine and their metabolites), antidepressants (Citalopram, Mirtazapine and their metabolites, Trazadone and its psychoactive metabolite meta-Chlorophenylpiperazine) and Gabapentin. Additional findings included samples positives for cocaine and its metabolites BZE and EME, cannabinoids, amphetamine and synthetic cathinone methylenedioxypyrovalerone and synthetic cannabinoids from JWH family.

In urine samples in which methadone was not found, screening analysis revealed the presence of the opiates (buprenorphine, 6-MAM, morphine, codeine, dextromethorphan), cocaine, cannabinoids and fentanyl and analogs.

### 2.3. Fentanyl, Fentanyl Analogs and Novel Synthetic Opioids

Toxicological screening analysis revealed the presence of fentanyl and analogs and/or metabolites in 23 (7.8%) out of 296 screened urine samples. No other NSOs were found.

In 4 out of 23 samples, the substances matched while in other cases, parent drug was identified by one method and metabolite by the other, or similar compounds were determined.

**Figure 2:**
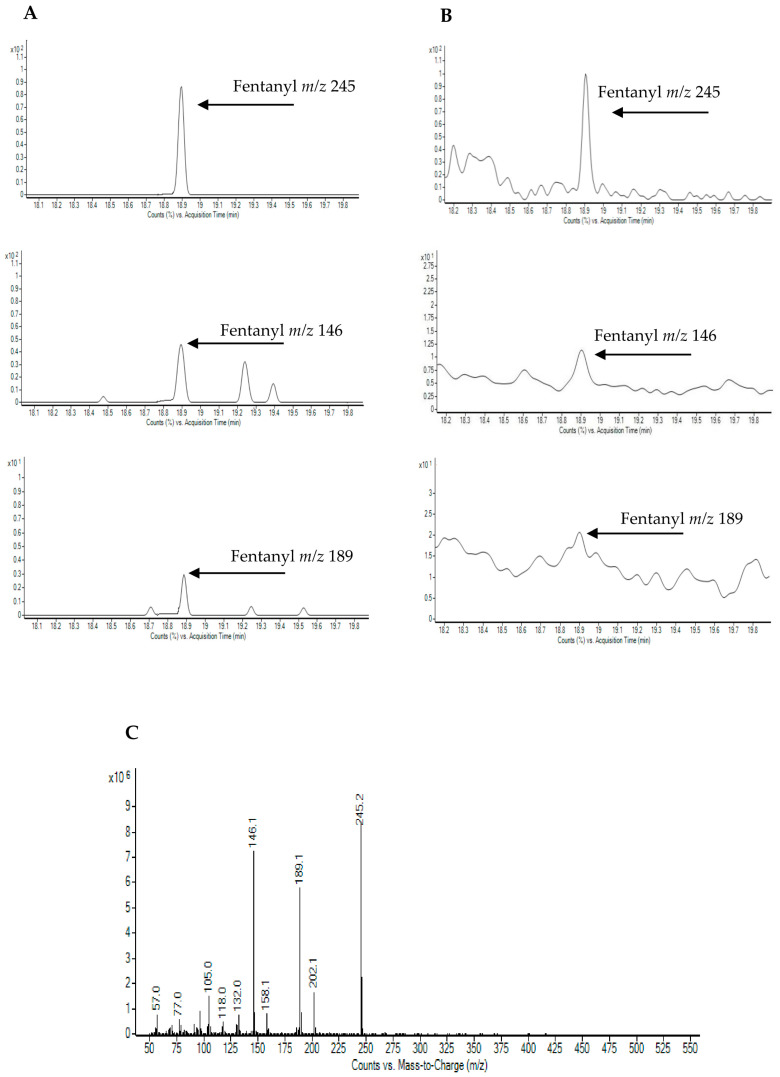
Representative selected ion monitoring GC-MS chromatograms of: urine samples positive to Fentanyl (**A**,**B**) and mass spectrometry or tandem mass spectrometry (MS/MS) full scan mass spectrum used for substance identification (**C**).

**Figure 3:**
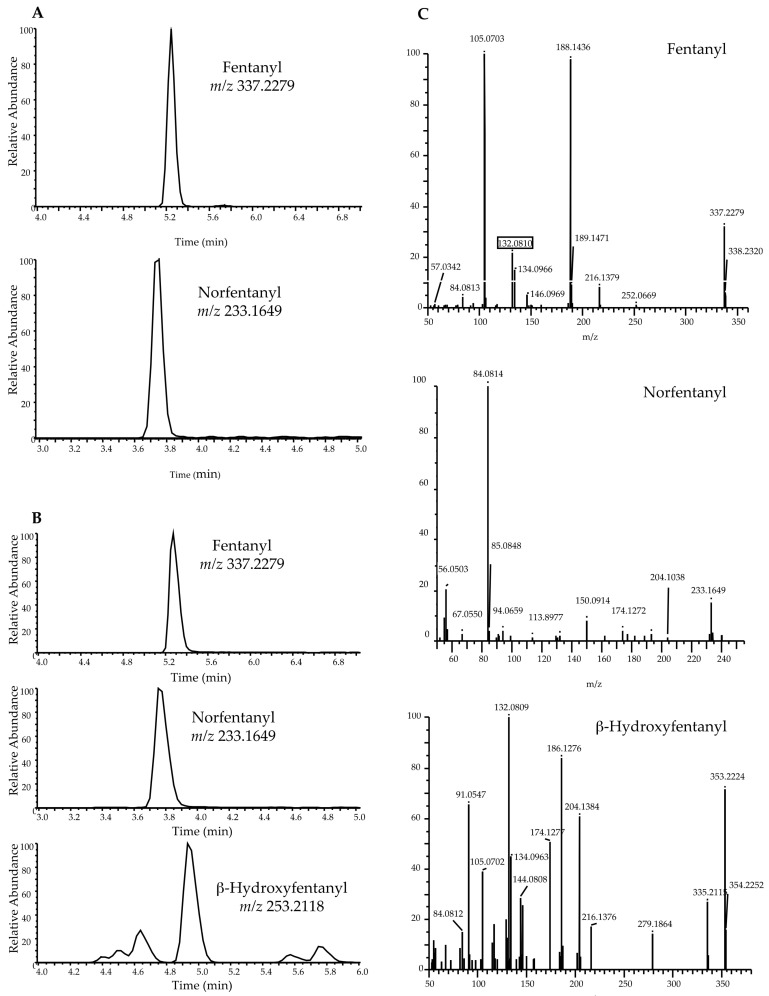
Representative extracted-ion UHPLC-HRMS chromatograms of: (**A**) urine sample positive to Fentanyl and Norfentanyl (**B**) urine sample positive to Fentanyl, Norfentanyl and β-hydroxyfentanyl and MS/MS full scan mass spectrum used for substances identification (**C**).

Chromatogram sin GC-MS and UHPLC-HRMS of 2 positive fentanyl samples are shown in Figure 2 and Figure 3, respectively, and screening results on fentanyl positive samples were reported in Table 2.

### 2.4. Other NPS

The NPS, other than NSOs, detected in the 296 analyzed samples by both methodologies belonged to the class of synthetic cathinones (4.4%) and to that of synthetic cannabinoids (1.3%) (Table 3).

**Figure 4:**
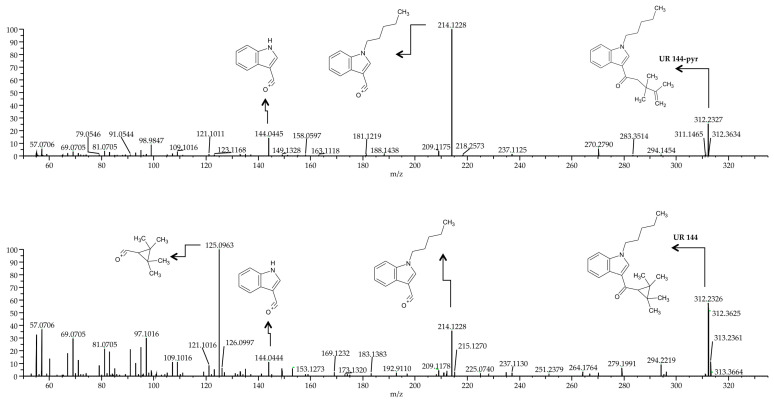
Full mass spectra of UR-144-pyr and UR-144.

The analysis by UHPLC-HRMS method of real sample obtained from the subject that results positive to UR-144 showed two peaks with different retention time but similar mass spectrum (Figure 4).

## 3. Discussion

Methadone is frequently prescribed for the maintenance therapy of opioid addiction detoxification. Patients needing treatment with this and other medications often have co-occurring medical and mental illnesses that require medication treatment [24].

Untargeted mass spectrometry techniques have become essential tools for toxicological analysis [25]. 

The poor availability of reference standards for many NPS and metabolites presents a large challenge to forensic toxicology laboratories when trying to detect and identify both known and unknown NPS and other xenobiotics. What toxicologists expect both in clinical and forensic analysis from a general unknown screening procedure is the unequivocal identification of the xenobiotics involved in intoxication cases, even when they have no evidences to guide the search. 

In general, the combination of different complementary methods (immunoassays, liquid chromatography and gas chromatography) was shown to be a good approach for screening samples in forensic and clinical toxicology [26]. Currently, the most competent approach for compound identification involves mass spectral library search [27].

We here presented two complementary analytical methods for screening of classic drugs of abuse and new psychoactive substances and metabolites in urine samples. Low resolution GC-MS and high-resolution instruments (UHPLC-HRMS) can both be used to develop efficient screening workflows. It was possible to obtain an identification, based on the obtained mass spectrometry information, of different xenobiotics.

The main purpose of this initial screening technique has been to identify samples positive to classical drugs of abuse, NPS and NSOs while simultaneously eliminating negative specimens from any subsequent analytical examination. Once a NPS or a NSO are detected, quantification could be further performed to provide information regarding concentrations found in urine of users and in cases of fatal and non-fatal intoxications.

The principal limitation of the presented methodology was the difficulty associated with data processing to get the information from single sample analysis that required qualified expertise. Moreover, in some cases it can be extremely difficult to chromatographically separate certain NPS to facilitate identification via mass spectrometry, such as in the case of isomers and isobaric compounds which display the same or significantly related chemical formulae [22,28,29,30,31,32]. 

In the current method and in agreement with a previous study [29] the isomers JWH-019 and JWH-122 as well the two metabolites of JWH-122 (JWH 122 N-4-Hydroxypentyl and N-5-Hydroxypentyl) and JWH-210 (JWH 210 N-4-Hydroxypentyl and N-5-Hydroxypentyl) were not distinguishable, since their masses and retention times matched. Otherwise, the isomers UR-144 and UR-144-pyr could be distinguished (Figure 4). Moreover, the opiate family contains a number of isobaric couples that can complicate the correct identification of e.g., morphine versus hydromorphone, or codeine versus hydrocodone. Other potential isobaric/isomeric interfering compounds that we found in our run were amitriptyline versus EDDP, and Tramadol versus O-desmethylvenlafaxine. Nevertheless, in our developed methodology, the above reported substances exhibited different retention times. 

Isomeric and isobaric substances require gas or liquid chromatographic conditions that enable adequate separation of the compounds prior to MS analysis or include other mass spectrometry data such as *m*/*z*, isotope pattern, retention time and fragmentation information [22,30,31,32].

However, even if the total analysis time was not short, this method could screen several psychoactive substances of different chemical structures in epidemiological studies aimed to disclose the use of compounds with a high risk of toxicity, leading to severe acute intoxications and overdoses. Moreover, High resolution full scan data also provides retrospective analysis for identifying previously unknown drugs of abuse [31].

Indeed, for this particular study, no reference standards were used, but only mass spectrometric libraries and the coupling of both methodologies. As above reported, positivity to a certain substance was only provided when both methodologies, independently run by different operators, matched with the identification of a specific molecule. 

In agreement with previous studies [15,19,21], the HRMS procedure was shown to be superior to screening by GC-MS, the costs still limit the widespread distribution in routine laboratories.

On the other hand, a last generation GC-MS assay highlighted the similar specificity of UHPLC-HRMS and therefore the simultaneous use of the two instruments allowed to demonstrate that a simple and traditional methodology can be used to screen unknown samples this also due to the presence of the latest generation of libraries present in support to toxicologist whose experience allows to identify unknown substances or to exclude false positives.

In this concern, analytical methodologies used for the identification of NPS continuously emerging in illicit markets should be developed, validated, updated and analytical data should always be shared across different communication platforms to help health professionals involved in clinical and forensic toxicology issue [6,33].

In addition, once substances identification has been accomplished, it can be of interest to confirm and quantify identified substances to expand information on concentration found in biological fluids of consumers and eventually associate obtained data with clinical evidence. In this concern, pure standards of parent compounds and/or metabolites are needed an extensive method validation whatever is the applied methodology (e.g., LC-MS/MS, GC-MS, GC-MS/MS or HRMS) considering the maximum cost-benefit ration for a high throughput laboratory facing with this kind of analyses.

## 4. Materials and Methods

### 4.1. Chemicals and Reagents

Water, methanol (MeOH) and acetonitrile (ACN) MS grade, chloroform, isopropanol and formic acid analytical grade were purchased by Carlo Erba (Milan, Italy). Ammonium formate, phosphate buffer and N,O-bis-trimethylsilyl-trifluoroacetamide (BSTFA) with 1% trimethylchlorosilane (TMCS) was obtained from Sigma–Aldrich (Milan, Italy).

### 4.2. Study Design

Urine samples collection took place at Consorcio Mar Parc De Salut De Barcelona, Spain and Hospital Universitari Germans Trias i Pujol from March, 2019 through October, 2020. Here, 296 patients with a history of opioid use disorder were enrolled in this study. All individuals were under methadone maintenance therapy (MTT). In this case, 109 patients provided identified urine samples after obtaining a signed informed consent, while 187 accepted to provide an anonymous sample, but no personal information was collected.

In order to secure the participants’ privacy, the survey data and collected urine were coded and the local Human Research Ethics Committee of both centers (ref. 2018/2138/I and PI-18-126) approved the study protocol. Prior to analysis aliquots of urine were stored at −20 °C.

### 4.3. Sample Preparation for Screening Analysis by GC-MS and UHPLC-HRMS

A liquid-liquid extraction was performed after diluting 0.5 mL of urine in 1 mL 0.1 M phosphate buffer pH 3.0 and 0.5 mL of the same sample in 0.1 M phosphate buffer pH 10 (the desired pH was eventually adjusted using drops of 1 N HCl or 1N KOH, respectively). The samples were vortex mixed and then the solutions were extracted twice with 1.5 mL chloroform/isopropanol (9:1, v:v). After centrifugation, the organic layer from each buffered sample was divided into two 1.5 mL aliquots and evaporated to dryness at 40 °C under a nitrogen stream.

The first dry aliquot was derivatized with a mixture of 25 μL of acetonitrile and 25 μL of N,O-bis-trimethylsilyl-trifluoroacetamide (BSTFA) with 1%trimethylchlorosilane (TMCS) at 70 °C for 30 min. The second dry aliquot was dissolved in 50 μL ethyl acetate. A 1 μL amount of underivatized and derivatized acid and alkaline extracts were injected into the GC-MS system.

After the analysis in GC-MS, the underivatized samples were evaporated to dryness under a nitrogen stream and then dissolved in 150 μL of a mixture of mobile phase A (Ammonium formate 2 mM, 0.1% HCOOH) and B (Ammonium formate 2 mM in MeOH/ACN 50/50, 0.1% HCOOH, 1% H2O) (50:50, *v*/*v*). 5 μL were injected into UHPLC-HRMS.

### 4.4. Gas Chromatography-Mass Spectrometry (GC-MS) Instrumentation

The GC-MS instrument consisted of an Agilent 7890 A gas chromatograph coupled with 5975 C mass spectrometry detector (Agilent Technologies, PaloAlto, CA, USA). Ultra-Inert GC column Zebron (ZB-Drug-1, 15m × 250 µm i.d, film thickness 0.25 µm; Phenomenex, Milan, Italy) was installed.

The GC-MS condition for the screening procedure was as follows: splitless injection mode; helium (purity 99%) carrier gas flow 1.2 mL/min; the injection port, ion source, quadrupole and transfer line temperatures were 260, 230, 150 and 320 °C, respectively; column temperature was programmed at 70 °C for 2 min and increased to 190 °C at 30 °C/min and then increased to 290 °C at 5 °C/min for 10 min. Subsequently the programed temperature was increased to 340 °C at 40 °C/min to eliminate impurities from the column.

The electron-impact (EI) mass spectra were recorded in total ion monitoring mode (scan range 40–550 *m*/*z*).

The full scan data files were processed by an Agilent Workstation (Agilent Technologies). The mass spectra international libraries used for peaks identification were NIST Research Library (National Institute of Standards and Technology)

### 4.5. Ultra-High-Performance Liquid Chromatography-High-Resolution Accurate Masses Spectrometry (UHPLC-HRMS) Instrumentation

The UHPLC/ESI Q-Orbitrap system consisted of an Ultimate3000 LC pump and an Ultimate 3000 autosampler coupled with a QExactive Focus mass spectrometer equipped with a heated electrospray ionization (HESI) probe operating in positive ionization mode and the system was controlled by Trace finder 4.0 software (Thermo Fisher Scientific, Bremen, Germany).

Separation was performed on an Accucore™ phenyl Hexyl (100 × 2.1 mm, 2.6 μm, Thermo, USA). They were maintained at 40 °C. The flow rate was set at 500 μL/min. Elution was achieved as follow: 99% A for 1 min, linear gradient to 99% B in 10 min, held for 1.5 min. The column re-equilibration was performed with a linear gradient to 99% A in 0.01 min, held for 4.0 min. A heated electrospray ionization (HESI) source in positive/negative ion mode was used for the ionization of compounds.

The mass parameters were as follows: ionization voltage was 3.0 kV; sheath gas and auxiliary gas were 35 and 15 arbitrary units, respectively; S-lens RF level 60; vaporizer temperature and capillary temperature were setting both at 320 °C. Nitrogen was used for spray stabilization, for collision induced dissociation experiments in the HCD cell and as the damping gas in the C-trap. The instrument was calibrated in the positive and negative modes every week.

Data were acquired in full-scan in data-dependent MS2 (ddMS2) mode. In this mode, both positive and negative high-resolution, full-scan data at resolution of 70 k were collected with a scan range of 100–1000 *m*/*z*, then MS2 spectra at a resolution of 17.5 k with an isolation window of 2 *m*/*z* were triggered for compounds entered in the inclusion list and expected retention times of the target analytes, with a 1 min time window.

The MS and fragmentation data acquired in full scan is processed by Thermo Scientific TraceFinder™ software. This specific software performs a thorough interrogation of the database by making use of the built-in database and mass spectral library of over 1400 compounds, retention times, isotope pattern matching, elemental composition determinations to identify and confirm drugs and metabolites in the analyzed samples. Moreover, mzCloud Mass Spectral Library was used as mass spectra international library for unknown peak identification (Advanced Mass Spectral Database; www.mzcloud.org, accessed on 1 April 2021).

### 4.6. Analytical Performance

To check the robustness and the reliability of the developed analytical methods, 10 different proficiency urine testing samples from UNODC International Quality Assurance Program (some with no analytes, some with one and some with more substances), whose previous qualitative and quantitative GC–MS results were available, were re-analyzed using the present methods.

Moreover, we also tested 10 urine samples fortified with 1 ng/mL 40 different most popular NPS and main metabolites prepared within the framework of an Italian Project (“NPS-LABVEQ” project) founded by Italian antidrug policy department aimed to allow pharmacotoxicological laboratories along the Italian peninsula to identify these substances in biological and non-biological matrices with different NPS [34]. Finally, 20 blank urine samples from laboratory personnel were also tested to check for false positives during the different batches.

## 5. Conclusions

This study presents a comprehensive gas chromatography-mass spectrometry (GC-MS) and liquid chromatography (UHPLC)–high-resolution mass spectrometry (HRMS) general screening procedure for classic drugs and new psychoactive substances in urine of consumers involving an easy, quick and low-cost sample preparation. This screening method based on two different chromatographic and mass spectrometry methodologies can be applied to disclose suspected drugs of abuse related fatalities or acute intoxications occurring in emergency departments and drug addiction services.

## Figures and Tables

**Table 1:** List of different target compounds, retention times (Rt) and monitored ions (*m*/*z*) using for the screening gas chromatography-mass spectrometry (GC/MS) and ultra-high-performance liquid chromatography-high-resolution mass spectrometry (UHPLC-HRMS) analysis.

Compound	Formula	GC/MS			UHPLC-HRMS		
		Rt (min)	Target *m*/*z* ion (Q)	Fragment *m*/*z* ions (Q/q) ^a^	Rt (min)	Target *m*/*z* ion [M+H]^+^ (Δ-error, ppm) ^b^	Fragment *m*/*z* ions
**Anticonvulsants**							
Carbamazepine	C_15_H_12_N^2^O	15.9	236	193(0.21) 165(1.4)	5.83	237.1022(−2.53)	194.0963 192.0805
Gabapentin	C_9_H_17_NO_2_	7.74	171	153(0.06) 110(0.13) 81(0.05)	2.79	172.1332(−3.48)	154.1227 137.0961 95.0860
Levetiracetam	C_8_H_14_N_2_O_2_	7.75	170	126(0.05) 98 (0.33) 69(0.13)	2.85	171.1128(−3.51)	154.0863 126.0914
Pregabalin	C_8_H_17_NO_2_	6.54	159	141(0.05) 103(0.03) 84(0.04)	2.61	160.1332(−3.75)	142.1227 97.1016 83.0861
Topiramate	C_12_H_21_NO_8_S	5.12	324	206(2.61) 189 (2.61) 127(1.62)	5.19	357.1326 * (2.20)	264.0532 184.0970 127.0391
**Antidepressants**							
Amitriptyline	C_20_H_23_N	9.59	277	215 (0.33) 202 (0.17) 58 (0.02)	5.98	278.19033 (−1.94)	233.1332 191.0861 105.0700
Bupropion	C_13_H_18_ClNO	17.53	239	139 (0.14) 100 (0.02) 44 (0.01)	4.52	240.1150 (−2.08)	184.0521 166.0419 131.0731
Citalopram	C_20_H_21_FN_2_O	17.27	324	238 (0.33) 208 (0.37) 58 (0.02)	5.44	325.1711(−1.59)	262.1026 234.0712 109.0452
Clomipramine	C_19_H_23_ClN_2_	17.22	314	268 (0.34) 85 (0.23) 58 (0.11)	6.23	315.1623(−1.59)	270.1044 86.0964 58.0651
Desmethylcitalopram	C_19_H_19_FN_2_O	17.42	310	238 (0.23) 138 (0.56) 44 (0.05)	5.40	311.1554(−1.60)	293.1446 262.1025 109.0451
Desmethylmirtazapine	C_16_H_17_N_3_	19.71	251	208 (2.50) 195 (0.08)	4.08	252.1495(−2.38)	235.1229 209.1073 195.0918
Mirtazapine	C_17_H_19_N_3_	19.51	265	208(0.39) 195(0.05) 167(0.5)	4.24	266.1652(−1.88)	209.1076 195.0917 72.0816
Trazodone	C_19_H_22_ClN_5_O	30.50	371	278(0.26) 205(0.05) 176(0.16)	4.95	372.1586(−1.34)	176.0819 148.0505 96.0446
**Antipsychotics**							
Levomepromazine	C_19_H_24_N_2_OS	19.32	328	282(6.34) 100(6.01) 58(0.79)	6.12	329.1682(−1.82)	242.0633 100.1126 58.0660
Norquetiapine	C_17_H_17_N_3_S	20.01	295	239(0.46) 227(0.09) 210(0.16)	5.26	296.1216(−1.69)	221.1080 210.0373 139.2405
Olanzapine	C_17_H_20_N_4_S	19.01	312	242(0.20) 229(0.25) 213(0.33)	3.09	313.1481(−1.92)	256.0901 213.0480 84.0814
Quetiapine	C_21_H_25_N_3_O_2_S	19.38	383	239(0.09) 210(0.04) 144(0.06)	5.61	384.1740(−1.56)	279.0949 253.0792 221.1071
Risperidone	C_23_H_27_FN_4_O_2_	8.1	410	233(0.09) 191(2.04) 177(1.30)	4.78	411.2191(−1.22)	191.1179 110.0600 69.0334
**Amphetamines**							
Amphetamine	C_9_H_13_N	5.40	135	91(0.04) 44(0.005)	2.84	136.1121(−3.67)	119.0857 91.0547
Ethylamphetamine	C_11_H_17_N	6.98	163	148 (0.11) 91 (0.02) 72 (0.005)	3.38	164.1434 (−3.05)	119.0858 91.0547
MDA	C_10_H_13_NO_2_	6.67	179	136(0.03) 77(0.08) 44(0.02)	3.24	180.1019(−3.33)	163.0753 135.0439 105.0699
MDMA	C_11_H_15_NO_2_	6.88	193	135(0.10) 77(0.83) 58(0.01)	3.31	194.1176(−2.58)	163.0753 135.04393 105.06986
Methamphetamine	C_10_H_15_N	5.80	149	134(0.25) 91(0.04) 58(0.01)	3.20	150.1277(−3.99)	119.0855 91.0541
**Benzodiazepines**							
7-Aminoclonazepam	C_15_H_12_ClN_3_O	12.60	285	256(1.15) 222(6.82) 194(6.82)	4.06	286.0742(−1.75)	250.0974 222.1025 194.0831
7-Aminoflunitrazepam	C_16_H_14_FN_3_O	11.33	283	264(5.00) 255(1.53) 240(5.55)	4.65	284.1194(−1.76)	227.0978 256.1243 148.0631
7-Aminonitrazepam	C_15_H_13_N_3_O	15.12	251	222(1.64) 195(5.55) 110 (5.55)	3.20	252.1131(−2.38)	224.1182 146.0714 121.0762
Alprazolam	C_17_H_13_ClN_4_	13.54	308	279(0.64) 245(2.29) 204(0.83)	6.38	309.0902(−1.62)	274.1208 241.0528 205.0747
Clonazepam	C_15_H_10_ClN_3_O_3_	12.34	315	288(1.14) 280(0.73) 234(1.14)	6.18	316.0484(−1.58)	302.0448 241.0521 214.0415
Clonazolam	C_17_H_12_ClN_5_O_2_	17.99	353	324 (0.60) 249 (1.00) 203 (0.82)	5.65	354.0752 (−1.69)	326.0563 319.1064
Diazepam	C_16_H_13_ClN_2_O	17.66	284	283(0.77) 256(0.59) 221(1.43)	6.83	285.0789(−2.10)	222.1150 193.0885 154.0417
Etizolam	C_17_H_15_ClN_4_S	18.01	342	313 (2.64) 266 (3.22) 137 (4.83)	6.54	343.0779 (−1.46)	314.0388 259.0216
Flubromazolam	C_17_H_12_BrFN_4_		370	341 (0.60) 222 (0.45) 195 (2.25)	6.22	371.0302 (−1.62)	343.0096 292.1105 237.0951
Flunitrazepam	C_16_H_12_FN_3_O_3_	22.31	313	312(0.71) 285(0.65) 266(0.95)	6.25	314.0936(−1.59)	300.0902 268.1003 239.0976
Flualprazolam	C_17_H_12_ClFN_4_		326	297 (0.55) 257 (2.75) 222 (0.61)		327.0806 (−2.14)	299.0625 292.1124 223.0662
Nitrazepam	C_15_H_11_N_3_O_3_	24.08	281	280 (0.44) 253 (0.64) 206(0.78)	5.96	282.0873(−2.12)	268.0842 236.0944 207.0918
Nordiazepam	C_15_H_11_ClN_2_O	18.66	270	242(1.04) 235(3.61) 207(4.87)	6.41	271.0633(−1.84)	208.0994 165.0214 140.0261
Oxazepam	C_15_H_11_N_2_O_2_Cl	16.70	286	268(0.06) 239(0.07) 205(0.06)	6.11	287.0581(−2.09)	241.0525 269.0475 104.0498
Temazepam	C_16_H_13_ClN_2_O_2_	19.93	300	273(0.35) 271(0.12) 256(0.86)	6.51	301.0738(−1.99)	283.0630 256.0715 255.0681
**Cocaine**							
Benzoylecgonine	C_16_H_19_NO_4_	15.15	289	168(0.27) 124(0.07) 105(0.22)	3.84	290.1387(−1.72)	168.1019 105.0335 82.0650
Cocaethylene	C_18_H_23_NO_4_	15.03	317	196(0.23) 82(0.11) 105(0.35)	4.72	318.1704(−0.31)	196.1330 82.0657 105.0341
Cocaine	C_17_H_21_NO_4_	14.27	303	272(2.00) 182(0.24) 82(0.17)	4.25	304.1543(−1.97)	182.1175 82.0657 105.0337
Ecgonine methyl ester	C_10_H_17_NO_3_	7.12	199	182(1.63) 94(0.39) 82(0.31)	0.6	200.1281(−2.99)	182.1177 150.0911 82.0658
**Cannabinoids**							
11-OH-THC	C_21_H_30_O_3_	15.91	330	300(0.74) 299(0.16) 41(1.86)	8.15	331.2267(−1.81)	313.2161 193.1224 105.0703
Cannabidiol	C_21_H_30_O_2_	16.42	314	246(0.53) 231(0.06) 193(0.75)	8.64	315.2319(−1.59)	193.1225 135.1169 93.0704
Cannabinol	C_21_H_26_O_2_	17.30	310	295(0.11) 238(0.79) 165(2.36)	8.88	311.2006(−1.61)	293.1901 241.1224 223.1118
Delta-9-tetrahydrocannabinol	C_21_H_30_O_2_	16.90	314	299(0.79) 271(1.66) 231(1.01)	9.02	315.2319(−1.59)	193.1223 123.0441 93.0701
THC-COOH	C_21_H_28_O_4_	17.20	344	329 (0.70) 299(0.41) 41(0.40)	8.26	345.2060(−1.74)	327.1953 299.2004 193.1223
**Fentanyls and NSOs**							
4-ANPP	C_19_H_24_N_2_	18.16	280	189(0.08) 146(0.07) 91(0.24)	5.04	281.2012(−2.13)	188.1435 134.0965 105.0703
Acetyl fentanyl	C_21_H_26_N_2_O	18.01	322	231(0.03) 188(0.08) 146(0.05)	4.89	323.2118 (−1.54)	188.1434 105.0703 132.0809
AH-7921	C_16_H_22_C_l2_N_2_O	11.22	329	172(0.20) 144(0.20) 126(0.05)	3.73	329.1182(−1.52)	284.0610 189.9555 172.0610
Alfentanil	C_21_H_32_N_6_O_3_	18.47	416	289(0.01) 268(0.03) 140(0.04)	5.35	417.2609(−1.20)	268.17651 197.1284 165.10223
Alpha-methylfentanyl	C_23_H_30_N_2_O	18.30	350	259(0.05) 146(0.20) 91(0.25)	5.50	351.2431(−1.42)	202.1588 119.0856 91.0546
Beta-Hydroxyfenatnyl	C_22_H_28_N_2_O_2_	17.52	352	245 (0.02) 189 (0.05) 146 (0.03)	4.90	353.2224 (−1.42)	204.1384 186.1276 132.0809
Carfentanil	C_24_H_30_N_2_O_3_	18.72	394	303(0.01) 187(0.05) 105(0.08)	5.60	395.2329(−1.52)	134.0965 105.0702 113.0600
Despropionyl *para*-fluorofentanyl	C_19_H_23_FN_2_	18.45	298	207(0.08) 164(0.08) 136 (0.40)	5.33	299.1918(−2.01)	188.1435 134.0966 105.0703
Fentanyl	C_22_H_28_N_2_O	18.89	245	189(2.77) 146(1.57) 105(4.27)	5.38	337.2279 (−0.30)	188.1436 105.0703 132.08010
Fluorofentanyl	C_22_H_27_FN_2_O	17.05	354	263(0.01) 207(0.04) 164(0.02)	3.55	355.2180(−1.69)	234.1289 188.1433 105.0699
Isotonitazene	C_23_H_30_N_4_O_3_	17.76	410	236 (0.40) 107 (0.12) 86 (0.01)	7.02	411.2391(−1.21)	250.1077 100.1109 72.0809
MT-45	C_24_H_32_N_2_	12.01	348	257(0.01) 165(0.17) 91(0.05)	4.03	349.2638(−1.72)	181.1011 169.1699 87.0916
N-methyl Norfentanyl	C_15_H_22_N_2_O	17.32	246	189(0.12) 96(0.08) 82(0.22)	4.20	247.1805(−2.02)	150.0915 98.0969 69.0707
Norfentanyl	C_14_H_20_N_2_O	17.90	232	175(0.09) 159(0.12) 83(0.05)	3.77	233.1649 (−2.14)	204.1038 150.0914 84.0814
Ocfentanil	C_22_H_27_FN_2_O_2_	17.34	370	279(0.01) 176(0.05) 105(0.05)	4.83	371.2129(−1.62)	188.1434 134.0966 105.0702
Remifentanil	C_20_H_28_N_2_O_5_	16.81	376	227(0.02) 212(0.02) 168(0.01)	4.48	377.2071(−1.33)	228.1230 146.0964 113.0600
Sufentanil	C_22_H_30_N_2_O_2_S	18.50	386	289(0.01) 140(0.03) 93(0.03)	5.97	387.2101(−1.29)	355.1838 238.1257 111.0266
Thienyl fentanyl	C_19_H_24_N_2_OS	17.99	328	179(0.20) 97(0.03) 82(0.04)	4.87	329.1682(−1.82)	97.0111 82.0657
U-47700	C_16_H_22_C_l2_N_2_O	10.80	329	172(0.05) 125(0.02) 84(0.01)	3.52	329.1182(−1.52)	284.0596 172.9579 81.0699
**Opioids and SOs**							
6-Monoacetylmorphine	C_19_H_21_NO_4_	18.83	327	268(0.92) 214(2.44) 162(4.40)	3.37	328.1543(−1.82)	268.1327 211.0753 165.0698
Buprenorphine	C_29_H_41_NO_4_	32.0	467	434 (0.33) 410(0.17) 378 (0.04)	5.72	468.3108(−1.28)	396.2165 84.0808 55.0544
Codeine	C_18_H_21_NO_3_	16.94	299	229(3.33) 214(5.00) 162(3.00)	2.88	300.1594(−1.28)	243.1012 215.1065 58.0659
EDDP	C_20_H_23_N	11.96	277	262(2.17) 220(3.09) 165(3.82)	5.61	278.1903(−1.43)	249.1509 234.1275 186.1275
EMDP	C_19_H_21_N	11.60	263	208(0.08) 130(0.17) 115(0.20)	5.95	264.1747(−1.89)	235.1355 234.1275 220.1121
Hydrocodone	C_18_H_21_NO_3_	16.01	299	284(7.80) 242(1.50) 185(2.44)	3.35	300.1594(−1.99)	283.175 133.0860 89.0602
Hydromorphone	C_17_H_19_NO_3_	16.35	285	229(3.12) 214(4.08) 200(5.30)	2.49	286.1438(−1.75)	185.0597 227.0699 199.0753
Methadone	C_21_H_27_NO	13.41	309	178(0.33) 165(0.25) 72(0.03)	6.15	310.2165(−1.93)	105.0338 265.1584 223.1116
Morphine	C_17_H_19_NO_3_	17.18	285	268(6.67) 215(2.50) 162(2.13)	1.91	286.1438(−1.75)	201.0912 229.0857 183.0807
Norcodeine	C_17_H_19_NO_3_	16.84	285	242(6.67) 215 (2.00) 148 (2.50)	2.91	286.1438(−1.74)	268.13263 215.10689 225.09088
Normorphine	C_16_H_17_NO_3_	16.12	271	201(0.02) 150(1.05) 148(1.33)	1.23	272.1281(−2.20)	254.1173 201.0916 121.0649
Noroxycodone	C_17_H_19_NO_4_	15.32	301	216(1.76) 201(4.14) 188(3.63)	3.17	302.1387(−1.65)	284.1281 227.0941 187.0754
Noroxymorphone	C_16_H_17_NO_4_	15.30	287	253(5.93) 202(1.63) 174(4.15)	1.78	288.1230 (−2.08)	270.1122 213.0783 173.0597
Oxycodone	C_18_H_21_NO_4_	15.83	315	258(4.42) 230(1.91) 187(7.64)	3.21	316.1543(−1.90)	298.1438 256.1330 241.1093
Oxymorphone	C_17_H_19_NO_4_	16.25	301	244(9.07) 216(2.62) 203(6.18)	2.24	302.1387(−1.65)	284.1278 242.1173 227.0934
Tramadol	C_16_H_25_NO_2_	14.41	263	188 (2.00) 135 (2.00) 58(0.13)	4.13	264.1958(−2.27)	58.0659
**Synthetic Cannabinoids**							
JWH 018	C_24_H_23_NO	8.55	341	284(1.50) 214(1.31) 127(0.82)	8.74	342.1852(−1.75)	214.1224 155.0605 144.0444
JWH 073	C_23_H_21_NO	6.98	327	284(1.62) 200(0.98) 127(0.84)	8.58	328.1696(−1.52)	230.1172 155.0489 125.0962
JWH 073 N-4-Hydroxybutyl	C_23_H_21_NO_2_	11.10	343	270(0.95) 144(1.11) 127(0.77)	7.32	344.1645(−1.74)	155.0490 127.1062
JWH 081	C_25_H_25_NO_2_	11.57	371	314(2.00) 214(1.43) 185(1.43)	8.92	372.1958(−1.61)	214.1223 185.0596 144.0443
JWH 081 4-Hydroxynaphtyl	C_24_H_23_NO_2_	12.44	357	300(1.32) 214(1.31) 171(1.48)	8.36	358.1802(−1.39)	214.1222 171.0438 144.0443
JWH 081 N-5-Hydroxypentyl	C_25_H_25_NO_3_	19.93	387	314(1.45) 230(1.50) 185(0.90)	7.70	388.1907(−1.55)	230.1172 185.0596 144.0443
JWH 122	C_25_H_25_NO	9.33	355	338(1.82) 298(1.38) 214(1.45)	8.91	356.2009(−1.40)	214.1223 169.0646 141.0697
JWH 122 N-4-Hydroxypentyl	C_25_H_25_NO_2_	13.26	371	284(0.66) 169(0.92) 144(0.96)	7.81	372.1958(−1.61)	169.0647 141.0698
JWH 122 N-5-Hydroxypentyl	C_25_H_25_NO_2_	15.87	371	284(1.57) 141(1.29) 115(1.56)	7.80	372.1958(−1.61)	169.0646 141.0697
JWH 210	C_26_H_27_NO	10.77	369	352(1.71) 312(1.64) 214(0.90)	9.21	370.2165(−1.62)	214.1223 183.0804 144.0443
JWH 210 N-4-Hydroxypentyl	C_26_H_27_NO_2_	14.56	385	298(0.64) 183(0.86) 144(0.90)	8.08	386.2115(−1.29)	183.0804 155.0854 144.0443
JWH 210 N-5-Hydroxypentyl	C_26_H_27_NO_2_	17.79	385	368(2.75) 230(3.24) 144(2.20)	8.06	386.2115(−1.29)	230.1172 183.0803 155.0853
UR 144	C_21_H_29_NO	9.94	311	296(0.98) 214(0.13) 144(0.40)	9.07	312.2322(−1.60)	214.1223 125.0962 97.1016
UR 144 N-5-Hydroxypentyl	C_21_H_29_NO_2_	10.70	327	231 (0.33) 230(0.001) 144(0.10)	7.85	328.2271(−1.83)	230.1172 125.0962 97.1016
XLR 11	C_21_H_28_FNO	10.73	329	314(0.90) 232 (0.09) 144(0.36)	8.64	330.2228(−1.51)	232.1129 125.0962 97.1016
XLR 11 N-4-Hydroxypentyl	C_21_H_28_FNO_2_	11.73	345	330(0.83) 248(0.11) 144(0.29)	7.57	346.2177(−1.44)	248.1077 144.0443 67.0550
AM-2201	C_24_H_22_FNO	10.35	359	342 (0.20) 284 (1.25) 232 (1.30)		360.1764	
**Synthetic Cathinones**							
MDPV	C_16_H_21_NO_3_	8.23	275	149(0.25) 126(0.01) 119(0.50)	4.35	276.1594 (−2.17)	126.1278 149.0232
4-MEC	C_12_H_17_NO	6.43	191	119(0.33) 91(0.17) 72(0.03)	3.66	192.1383 (−2.60)	174.1277 159.1040 119.0857
Butylone	C_12_H_15_NO_3_	8.72	221	149(0.10) 121(0.20) 72(0.02)	3.52	222.1125(−2.25)	204.1018 174.0913 72.0815
Mephedrone	C_11_H_15_NO	6.45	177	119(0.20) 91(0.10) 58(0.02)	3.37	178.1226(−3.36)	160.1121 145.0886 119.0857
Methcathinone	C_10_H_13_NO	5.98	163	105(0.20) 77(0.07) 58(0.02)	2.67	164.107(−1.83)	146.0965 131.0731 105.0703
Pentylone	C_13_H_17_NO_3_	8.13	235	149(0.17) 121(0.25) 86(0.01)	4.16	236.1281(−2.54)	218.1174 188.1069 86.0969
**Miscellaneous**							
4-FA	C_9_H_12_FN	4.81	153	109(0.06) 83(0.10) 44(0.01)	2.95	154.1027(−3.24)	109.0451 137.0761 114.0917
4-MA or PMA	C_10_H_15_NO	9.50	165	122(0.03) 78(0.08) 44(0.01)	3.27	166.1226(−3.61)	150.0499 137.0419 117.0701
PMMA	C_11_H_17_NO	10.33	179	121(0.10) 78(0.13) 58(0.01)	3.43	180.1383(−2.78)	149.0961 121.0649
*m*-CPP	C_10_H_13_ClN_2_	7.39	196	154 (0.25) 138 (2.00)	3.97	197.0840(−3.04)	154.0416 119.0730
Ketamine	C_13_H_16_ClNO	8.28	237	209(0.07) 179(0.02) 125(0.08)	3.77	238.0993(−2.51)	207.0574 179.0622 125.0154

a, Q/q ion abundance ratio; b, delta error (ppm); * Sodium adduct; MDA: 3,4-Methylenedioxyamphetamine; MDMA: 3,4-Methylenedioxymethylamphetamine;11-OH-THC: 11-Hydroxy-delta-9- tetrahydrocannabinol; THC-COOH: 11-nor-9-carboxy-delta-9- tetrahydrocannabinol carboxilc acid; NSOs: novel Synthetic Opioids;4-ANPP: 4-Aminophenyl-1-phenethylpiperidine or Despropionyl fentanyl; AH 7921: 3,4-dichloro-N-[[1-(dimethylamino)cyclohexyl]methyl]-benzamide;U-47700: *trans*-3,4-dichloro-N-[2-(dimethylamino)cyclohexyl]-N-methyl-benzamide; MT-45: 1-cyclohexyl-4-(1,2-diphenylethyl)-piperazine, dihydrochloride; SO: Synthetic Opioids; EDDP: 2-Ethylidene-1,5-dimethyl-3,3-diphenylpyrrolidine; EMDP: 2-ethyl-5-methyl-3,3-diphenylpyrroline; MDPV: 3,4-Methylendioxy Pyrovalerone; 4-MEC: 4-Methylethcathinone;4-FA: 4-Fluoroamphetamine; 4-MA or PMA: 4-Methoxyamphetamine or *para*-methoxymethylamphetamine; PMMA: *para*-methoxymethamphetamine; *m*-CPP: 1-(3-Chlorophenyl)piperazine, SO: synthetic opioids.

**Table 2:** Comparison of GC/MS and UHPLC-HRMS fentanyl and/or its metabolites and analogs urine sample screening and confirmation results.

Sample Code	Detected Compound (GC/MS)	Detected Compound UHPLC-HRMS
MI-1029	ND	Fentanyl Norfentanyl
MI-1077	Fluorfentanyl	ND
MI-1078	N-(3-ethylindole) Norfentanyl	ND
MI-1079	Fluorfentanyl	Fentanyl Beta-Hydroxyfentanyl Norfentanyl
BS-2003	Fentanyl	Fentanyl
MI-3009	Fluoro acetyl Fentanyl	ND
MI-5016	Fluoro Valeryl fentanyl	ND
US-010	Fentanyl	Fentanyl Norfentanyl
US-017	Fentanyl	Norfentanyl
US-039	Fentanyl	Beta-hydroxyfentanyl Fentanyl Norfentanyl
US-059	Fluorfentanyl	ND
US-060	ND	Norfentanyl
US-065	Fluorfentanyl	ND
US-077	Fluoro Valeryl fentanyl	ND
US-083	Fluoro Valeryl fentanyl	ND
US-095	Fluoro Valeryl fentanyl	ND
US-109	Fluoro Valeryl fentanyl	ND
US-139	Acetyl-methylfentanyl 2’-fluoro ortho-Fluorofentanyl	ND
US-142	Thiofentanyl	ND
US-144	Fentanyl	Fentanyl Norfentanyl Beta-Hydroxyfentanyl
US-145	Fluorfentanyl	ND
US-148	Fentanyl	Norfentanyl
US-155	Thiofentanyl	Norfentanyl

ND: not detected.

**Table 3:** New psychoactive substances(NPS) found in urine samples under investigation.

NPS Classes	Substances (*n*)
synthetic cathinones	MDPV (2) 4-cloro N butylcathinone (1) 4-Methyl-PV8 (6) Fenethylline (4)
synthetic cannabinoids	JWH-122 (1) JWH-032 (1) JWH-200 (1) UR-144 (1)

*n* = number of positive samples; MDPV: 3,4-Methylendioxy Pyrovalerone; 4-Methyl-PV8: 2-(pyrrolidin-1-yl)-1-(p-tolyl)heptan-1-one.

## Data Availability

All data generated or analyzed during this study are included in this published article.
